# Multi-Wavelength Autofluorescence Characteristics and Association With Inflammation in Acute Posterior Multifocal Placoid Pigment Epitheliopathy

**DOI:** 10.1167/iovs.66.11.62

**Published:** 2025-08-26

**Authors:** Robert P. Finger, Lennart J. Overbeck, Moritz Berger, Marie D. Just, Jana K. Koch, Jan H. Terheyden, Selina Foti, Matthias Schmid, Frank G. Holz, Thomas Ach, Maximilian W. M. Wintergerst

**Affiliations:** 1 Department of Ophthalmology, University Hospital Bonn, Venusberg-Campus 1, Bonn, Germany; 2 Department of Ophthalmology, University Medical Center Mannheim, Heidelberg University, Mannheim, Germany; 3 Department of Medical Biometry, Informatics and Epidemiology, University of Bonn/University Hospital Bonn, Bonn, Germany; 4 Core Facility Biostatistics, Central Institute of Mental Health, Medical Faculty Mannheim, Heidelberg University, Mannheim, Germany; 5 Augenzentrum Grischun, Chur, Switzerland

**Keywords:** acute posterior multifocal placoid pigment epitheliopathy (APMPPE), inflammatory activity, imaging biomarker, phenotyping, multimodal imaging

## Abstract

**Purpose:**

The purpose of this study was to investigate characteristics of chorioretinal lesions secondary to acute posterior multifocal placoid pigment epitheliopathy (APMPPE) using multi-wavelength fundus autofluorescence (FAF) and their association with intraocular inflammation.

**Methods:**

In this exploratory cross-sectional study, patients with chorioretinal lesions secondary to APMPPE underwent multimodal imaging including FAF with 450 nm, 488 nm, 518 nm, and 787 nm excitation wavelength, color fundus photography (CFP), and optical coherence tomography (OCT). Lesions were graded for FAF and CFP characteristics and inflammatory activity by an experienced image grader and an ophthalmologist. Association between these parameters was assessed using binary and ordinal regression models.

**Results:**

Twenty-eight eyes (15 patients) with 597 lesions were included. Inter-reader reliability was almost perfect for qualitative image analysis and moderate for clinical activity grading. Lesions detectable on FAF were most often invisible (44.7%), followed by greyish/brownish in color (34.1%) on CFP. Invisible lesions on CFP were most frequently hypo-autofluorescent, whereas greyish/brownish colored lesions on CFP were most frequently hyper-autofluorescent on FAF. Ninety-nine percent of the lesions invisible on CFP were evident on 787 nm-excitation wavelength FAF (787 nm FAF). For multiple CFP categories, hyper- and iso-autofluorescence was more frequent on shorter wavelengths, and hypo-autofluorescence was more frequent on longer FAF excitation wavelengths. Hyper-autofluorescent lesions were more likely to be clinically active as compared to hypo-autofluorescent lesions (probabilities ranging between 0.85–0.87 and 0.51–0.61).

**Conclusions:**

FAF can aid phenotyping APMPPE lesions and assessing inflammatory activity and should therefore be routinely included in clinical management and studies. Especially 787 nm FAF allows visualization of APMPPE lesions not visible on clinical examination or CFP and could therefore be a valuable addition to routine imaging protocols.

Acute posterior multifocal placoid pigment epitheliopathy (APMPPE) is a white-dot syndrome usually affecting healthy young adults between the age of 20 and 50 years. Patients suffer acute, mostly bilateral, painless loss of vision. Funduscopic examination shows characteristic multifocal, yellow-white, placoid chorioretinal lesions.^1^^–^^4^ Although the disease was first thought to affect mostly the retinal pigment epithelium (RPE), choroidal involvement prior to RPE affection has been suggested since.^5^^–^^8^ Etiology of APMPPE remains unclear. Anecdotal evidence suggests prior viral infections as a possible trigger of the inflammatory episode.^1^^,^^2^ In some cases, affiliation with systemic diseases, such as cerebral vasculitis, has been described.^9^^,^^10^ Whereas most patients have a favorable prognosis, studies show that a significant proportion of patients experience an incomplete or limited visual recovery with persisting visual symptoms and considerable visual loss in some cases.^11^^,^^12^ To date, no consensus on the need for treatment has been reached.^9^^,^^11^^,^^13^

Multimodal imaging has become a corner stone in the APMPPE diagnostic workup and aided in better understanding this disease. Multimodal imaging apart from fundus autofluorescence (FAF) includes fluorescein angiography (FA), indocyanine green angiography (ICGA), optical coherence tomography (OCT), and OCT angiography.^4^^,^^7^^,^^14^^–^^18^ FAF offers a broad spectrum of excitation wavelengths ranging from 450 nm excitation FAF (450 nm FAF), 488 nm excitation FAF (488 nm FAF), 518 nm excitation FAF (518 nm FAF) to 787 nm excitation FAF (787 nm FAF). Different excitation wavelengths result in excitation of different ocular fluorophores and different penetration depths.^19^ On a cellular level, short-wavelength excitation primarily leads to fluorescence of lipofuscin granules in the RPE.^20^^,^^21^ The autofluorescence generated by 787 nm FAF on the other hand has mainly been attributed to melanin not only in the RPE but also the underlying choroid.^22^^,^^23^ The main source of 787 nm FAF signal on a subcellular level was suggested to be melanin in melanosomes and melanolipofuscin granules.^24^

FAF imaging contributes to the understanding of various retinal diseases, such as age-related macular degeneration (AMD) or hereditary retinal diseases.^25^ Furthermore, studies have shown the added value in using multiple excitation wavelengths. In AMD, for example, 450 nm FAF and 518 nm FAF have been used to quantify geographic atrophy, whereas also 787 nm FAF can provide additional pathophysiologic insights.^26^^–^^29^ The 787 nm FAF was further deemed useful for monitoring patients with retinitis pigmentosa, with authors emphasizing the increased patient comfort compared to imaging with 488 nm FAF.^30^^,^^31^

Studies comparing multiple FAF wavelengths have been conducted for different subtypes of posterior uveitis, such as punctuate inner choroidopathy (PIC) or multiple evanescent white-dot syndrome (MEWDS), and others.^18^^,^^32^^,^^33^ In contrast, publications considering FAF in APMPPE have mainly utilized 488 nm FAF, except one study where 585 nm excitation FAF was used.^34^ More recently, 450-nm Color-FAF was introduced as a tool to distinguish APMPPE and other posterior uveitis entities. This novel modality utilizes a single excitation wavelength, with the emitted fluorophores being spectrally resolved allowing separate detection of green and red components.^35^

Most authors describe lesions in the acute phase of APMPPE as hypo-autofluorescent (hypo-AF) with a surrounding hyperautofluorescent (hyper-AF) border. In subacute lesions, hyper-AF becomes more prominent, resulting in a mixed pattern of hyper- and hypo-AF. Over time, areas of hypo-AF persist corresponding to regional RPE atrophy.^6^^,^^7^^,^^14^^,^^17^^,^^18^^,^^36^^–^^38^

For standardized use of multiple-wavelength FAF in clinical monitoring, therapeutic management, and clinical studies in APMPPE, relevant questions considering lesion characteristics and their association with inflammatory activity need to be answered. As a systematic analysis including multiple FAF excitation wavelengths is yet to be conducted, we assessed the characteristics of chorioretinal APMPPE lesions on four different FAF excitation wavelengths and their association with characteristics on color fundus photography (CFP) and inflammatory activity.

## Methods

### Subject Recruitment and Clinical Assessment

This cross-sectional study included patients with APMPPE according to the diagnostic criteria defined by the Standardization of Uveitis Nomenclature working group.^4^ Patients were recruited from uveitis and other outpatient clinics at the Department of Ophthalmology at the University of Bonn, Germany, between December 2017 and June 2021. All subjects gave their written informed consent after explanation of the nature and possible consequences of the study prior to being included. Ethics approval was obtained by the ethics committee at the University of Bonn (ethics approval ID 011/18), and the study was conducted in adherence to the Declaration of Helsinki. For each subject, clinical and demographic parameters were collected from medical charts. Best-corrected visual activity was assessed using LogMAR charts. Clinical grading of intraocular inflammation included anterior chamber (AC) cells and vitreous cells (according to the National Institutes of Health [NIH] grading system) and followed Standardization of Uveitis Nomenclature (SUN) recommendations.^39^^,^^40^ OCT images were analyzed for the presence of macular edema (ME). The ME was categorized into three categories: absence of ME (0), presence of intraretinal or subretinal fluid but no change in macular contour (1), and presence of intraretinal or subretinal fluid with change of macular contour (2) (Supplementary Fig. S1).^41^

### Image Acquisition and Analysis

Eyes were imaged using macula-centered CFP (confocal scanning laser ophthalmoscopy = 60 degrees  ×  55 degrees, image resolution = 3680  ×  3288 pixels; EIDON, CenterVue, now iCare, Padova, Italy). The corresponding fundus areas were then imaged using greyscale FAF with 450 nm (60 degrees× 55 degrees, image resolution = 3680 × 3288 pixels; EIDON, CenterVUE, now iCare, Padova, Italy),^26^ 488 nm, 518 nm, and 787 nm excitation wavelength (55 degrees widefield lens, image resolution = 1536 × 1536 pixels; Spectralis HRA, Heidelberg Engineering Inc., Heidelberg, Germany). Spectralis FAF images were acquired in high-resolution (HR) mode, with an automatic real-time tracking (ART) value of 100. FAF and CFP images were exported as PNG for further analysis. In addition to FAF imaging, OCT scans were obtained using Spectralis OCT (Heidelberg Engineering Inc., Heidelberg, Germany). For each patient, a 30 degrees or 55 degrees fovea-centered scan with an ART value of minimum 40 and minimum 61 B-scans was acquired. FA and ICGA images were not part of the standard imaging protocol, but available in a part of the cohort due to other reasons.

The CFP and four different FAF images from each eye were processed using the GNU Image Manipulation Program (GIMP).^42^ Each image modality was assigned its own layer, and the images were then re-scaled for alignment. This enabled the readers to easily perform intermodal cross-sectional comparisons by switching between image layers. Images were analyzed on a 27-inch 1920 × 1080 pixel Full HD monitor. Qualitative analysis of lesion characteristics was performed using GIMP with a zoom level of 25%. Qualitative image analysis and clinical activity grading were performed by an ophthalmologist (author M.D.J.) and an experienced image grader (author L.J.O.) specifically trained and supervised for these analyses by a senior ophthalmologist (author M.W.M.W.). In case of discrepancies, the grading of the trained grader was used for further analysis. Before analysis, every lesion visible on at least one modality was assigned a separate lesion ID by a single grader (author L.J.O.). All modalities were used to differentiate lesions. If lesions were difficult to differentiate, due to close proximity to each other or poorly defined borders, IDs were assigned based on the modality, where the lesions were most easily separable. In difficult cases, inter-rater agreement was qualitatively assessed and a senior ophthalmologist was consulted (author M.W.M.W.) to determine lesion borders and numbering, in order to guarantee comparability of the results in the following analysis (Supplementary Fig. S2). The first step of analysis was to determine which lesions were visible on the different modalities. For this, the images were viewed masked without knowledge of the assigned lesion number, starting with CFP. Afterward, the lesions were categorized by their characteristics using defined standardized descriptors/categories for lesion characteristics. In the CFP images, the following categories were utilized: dark (for highly pigmented lesions), not visible, white atrophic (for areas of atrophy without pigmentation), slightly visible or greyish/brownish (referred to as “greyish/brownish” in the following segments), and complex. Lesions were graded as complex if they simultaneously included at least one dark and one white atrophic area. On FAF images, the following categories were utilized: hypo-AF, iso-autofluorescent (iso-AF) and hyper-AF. All lesions containing hyper-AF parts were included in the hyper-AF category, meaning that in some cases these lesions could also include hypo-AF parts. Lesions, which were not assessable because they were cut off by image borders, covered by vitreous opacities, or in insufficiently illuminated image areas, were excluded from the analysis. Lesion activity was evaluated by clinical grading. As previously described in the literature, characteristics of active lesions included “creamy” lesion appearance, border “fluffiness” or “fuzziness,” bleedings, or edema.^1^^,^^2^^,^^16^^,^^43^^,^^44^ In questionable cases, the acquired OCT images were included in the evaluation as well as additional parameters from the clinical examination and FA or ICGA images, if available. Lesion activity was graded as either active or inactive.

### Statistical Analysis

Statistical analysis was conducted using R software (R: A Language and Environment for Statistical Computing, R Core Team, R Foundation for Statistical Computing; version 4.4.2, 2024). Weighted Cohens kappa (κ) was calculated to determine inter-reader reliability,^45^^,^^46^ based on mixed-effects cumulative and logistic regression models, as described by Wang et al.^47^ The κ values were interpreted using the following threshold: < 0.20 is considered poor agreement, 0.21 to 0.40 is considered fair agreement, 0.41 to 0.60 is considered moderate agreement, 0.61 to 0.80 is considered substantial agreement, and > 0.80 is considered almost-perfect agreement.

A mixed-effects cumulative logistic regression model (proportional odds model), including a random intercept for each patient and each eye nested within patients, was used to predict probabilities for different FAF characteristics (ordinal outcome with categories hypo-AF < iso-AF < hyper-AF) in dependence of CFP characteristics and FAF wavelength (predictors). Second, a mixed-effects logistic regression model, including a random intercept for each patient and each eye nested within patients, was used to predict probabilities of active inflammation (binary outcome) in dependence of FAF characteristics and FAF wavelength (predictors). In this analysis, only a subgroup of eyes with both active and inactive lesions were included to avoid perfect prediction in some study eyes. For both models, predicted probabilities were computed conditioned on the fixed effects, only (level-0 predictions). Subsequently, pairwise comparisons between predictor categories applying Bonferroni-Holm adjustments for multiple testing were performed in both cases. Any *P* < 0.05 was considered statistically significant.

## Results

### Characteristics of the Study Population

The characteristics of the study population are summarized in Table 1. Twenty-eight eyes of 15 patients diagnosed with APMPPE were included in the analysis of lesion characteristics on CFP versus FAF. Thirteen patients were bilaterally affected and two were unilaterally affected. All 28 eyes were phakic and showed no AC cells. Vitreous cells were found in 4 of the 28 eyes, the remaining 24 eyes showed no vitreous cells. ME of grade 1 was found in 5 of 28 eyes. The remaining eyes did not show any ME. Overall, 597 chorioretinal lesions were analyzed, 11 lesions were excluded from analysis according to the exclusion criteria. Fourteen eyes featured active APMPPE lesions (190 lesions were graded as active). The activity analysis included 14 eyes of 9 patients with 324 lesions, with 7 lesions excluded from this analysis. FA was available in 10 of 28 eyes overall and 10 of 14 eyes included in the activity analysis. ICGA was available in 8 of 28 eyes overall and 8 of 14 eyes included in the activity analysis. Inter-reader reliability was almost perfect for qualitative image analysis (Weighted Cohens kappa = 0.84, 95% confidence interval = 0.82–0.87) and moderate for clinical activity grading (Weighted Cohens kappa = 0.45, 95% confidence interval = 0.38–0.51).

**Table 1.** Characteristics of the Sample

	Mean (Range) ± SD or *N* (%)	
	Characteristics on CFP Vs. FAF Analysis	FAF Vs. Clinical Activity Analysis
Age, y	39.3 ± 16.5 (23–74)	43.6 ± 18.3 (23–74)
F	10 (66.6%)	5 (55.5%)
Duration of disease, mo	60.8 ± 96.8 (0–307)	4.9 ± 6.6 (0–23)
Current BCVA (log MAR)	0.07 ± 0.14 (0–0.6)	0.03 ± 0.05 (0.0–0.1)
Lesions per eye	21.3 ± 18.4 (1–72)	23.1 ± 11.2 (2–46)

BCVA, best corrected visual acuity; SD, standard deviation.

Inclusion criteria for subgroups are named in the “statistical analysis” section.

### Frequencies of Lesion Characteristics on CFP and FAF

Lesions on CFP were not visible in 44.7%. If visible, lesions were greyish/brownish in color (34.1%), white atrophic (13.3%), or complex (7.8%). No lesions were graded as dark on CFP. Lesions on FAF overall were most frequently hypo-AF (56.0%), followed by hyper-AF (23.4%) and iso-AF (20.6%).

### Association of Lesion Characteristics on FAF With Lesion Characteristics on CFP

Lesions which were not visible, white atrophic, or complex on CFP were most frequently hypo-AF on FAF, whereas greyish/brownish colored CFP lesions were most frequently hyper-AF on FAF. Lesions not visible on CFP were visible on FAF in 50%, 71%, 81%, and 99% of cases on FAF modalities (450 nm, 488 nm, 518 nm, and 787 nm FAF), respectively (Table 2). These tendencies were also evident in statistical evaluation of autofluorescence-characteristics (Supplementary Table S1). Please note that the values given in Table 2 correspond to the absolute/relative frequencies on an individual level, whereas the predicted probabilities in Supplementary Table S1 are on a marginal/population-average level.

**Table 2.** Characteristics on FAF in Relation to CFP Characteristics

CFP Category	FAF Category	450 nm FAF	488 nm FAF	518 nm FAF	787 nm FAF
Not visible	Hypo-AF	107 (40.8%)	163 (62.2%)	192 (73.3%)	238 (90.8%)
	Iso-AF	130 (49.6%)	75 (28.6%)	47 (17.9%)	3 (1.1%)
	Hyper-AF	25 (9.5%)	24 (9.1%)	23 (8.8%)	21 (8.0%)
White atrophic	Hypo-AF	38 (48.7%)	55 (70.5%)	57 (73.0%)	65 (83.3%)
	Iso-AF	22 (28.2%)	8 (10.2%)	5 (6.4%)	1 (1.2%)
	Hyper-AF	18 (23.0%)	15 (19.2%)	16 (20.5%)	12 (15.3%)
Complex	Hypo-AF	33 (71.7%)	36 (78.2%)	36 (78.2%)	36 (78.2%)
	Iso-AF	8 (17.3%)	3 (6.5%)	1 (2.1%)	0 (0.0%)
	Hyper-AF	5 (10.9%)	7 (15.2%)	9 (19.5%)	10 (21.7%)
Greyish/brownish	Hypo-AF	37 (18.5%)	48 (24.0%)	60 (30.0%)	111 (55.5%)
	Iso-AF	66 (33.0%)	56 (28.0%)	44 (22.0%)	14 (7.0%)
	Hyper-AF	97 (48.5%)	96 (48.0%)	96 (48.0%)	75 (37.5%)

AF, autofluorescent; CFP, color fundus photography; FAF, fundus autofluorescence.

Absolute values (relative values for one autofluorescence modality).

### Comparison of Characteristics Between Different Autofluorescence Modalities

**Figure 1.**
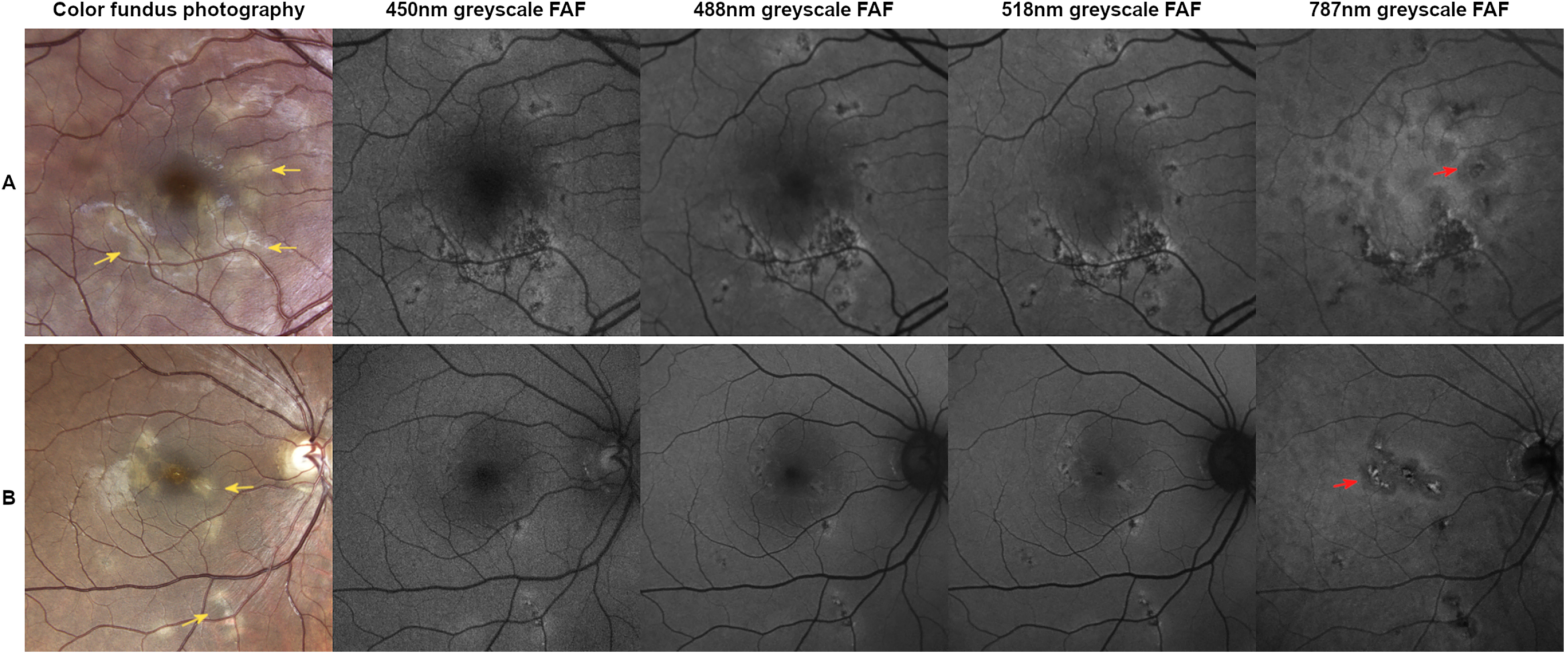
Comparison of inflammatory active lesions on color fundus photography (CFP) and multiple excitation wavelength fundus autofluorescence (FAF) imaging. Brightness levels of FAF images were adjusted for better comparison. *Row A* = a 31-year-old man with 6 months of disease duration, *Row B* = a 44-year-old man with 1 month of disease duration. Active lesions appear “fuzzy” with poorly defined borders on CFP (*yellow arrows*). On FAF, they are hypoautofluorescent with hyperautofluorescent borders. The area of hyperautofluorescence steadily decreases while the area of hypoautofluorescence steadily increases with longer FAF excitation wavelengths. The 787 nm FAF reveals a greater, hypoautofluorescent extent of lesion boundaries (*red arrows*) compared with the other modalities.

**Figure 2.**
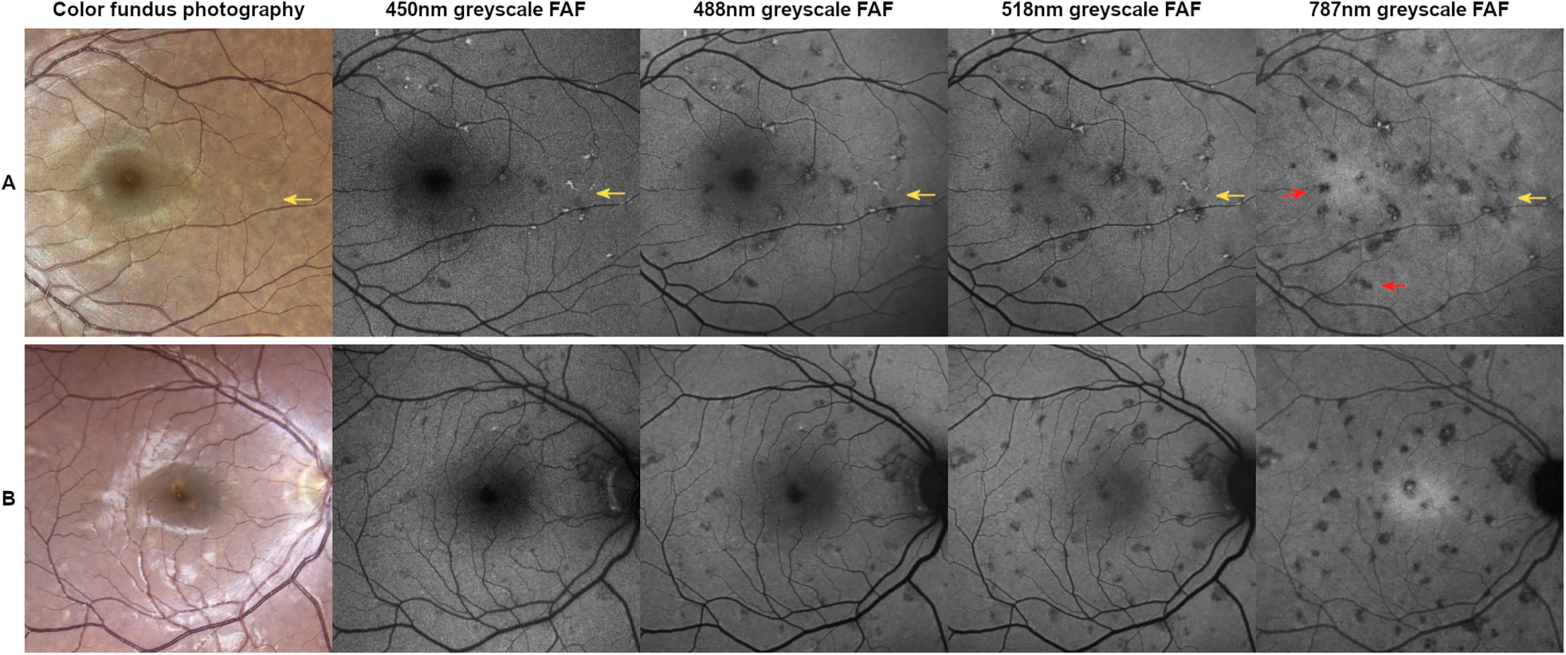
Comparison of lesions on color fundus photography (CFP) and 450 nm, 488 nm, 518 nm, and 787 nm excitation wavelength fundus autofluorescence (FAF) imaging. Brightness levels of FAF images were adjusted for better comparison. *Row A* = a 44-year-old woman with 3 months of disease duration. Many of the greyish/brownish CFP lesions are hyper-autofluorescent on FAF, especially on shorter excitation wavelengths (*yellow arrows*). On long excitation wavelength FAF, many lesions which are invisible on CFP are hypo-autofluorescent (*red arrows*). *Row B* = a 36-year-old man with 241 months of disease duration. Lesions not visible on CFP appear hypoautofluorescent on FAF to a great extent, especially on longer wavelengths.

Lesion characteristics on FAF followed steady significant trends in dependence of excitation wavelength for not visible, white atrophic, or greyish/brownish lesions: the frequency of hypo-AF lesions steadily increased on longer excitation FAF wavelengths, whereas frequency of iso- and hyper-AF lesions steadily decreased on longer excitation FAF wavelengths (see Table 2, Supplementary Table S2; Figs. 1, 2). In many cases, 787 nm FAF showed a greater extent of lesion boundaries compared with 450 nm, 488 nm, and 518 nm FAF (see Fig. 1).

### Comparison of Characteristics Between Different Color Fundus Photography Categories

The most pronounced differences in autofluorescence probabilities between two CFP categories were found when comparing greyish/brownish to not visible CFP lesions (Supplementary Table S3). Greyish/brownish colored lesions were more likely iso- and hyper-AF compared with not visible lesions across all FAF wavelengths. In reverse, not visible lesions were more likely hypo-AF compared with greyish/brownish colored lesions. The named differences were increasingly more evident on longer excitation FAF wavelengths (see Fig. 2). Further comparisons between other CFP categories are found in the supplementary materials.

### Association of Inflammatory Activity With Autofluorescence Characteristics

**Figure 3.**
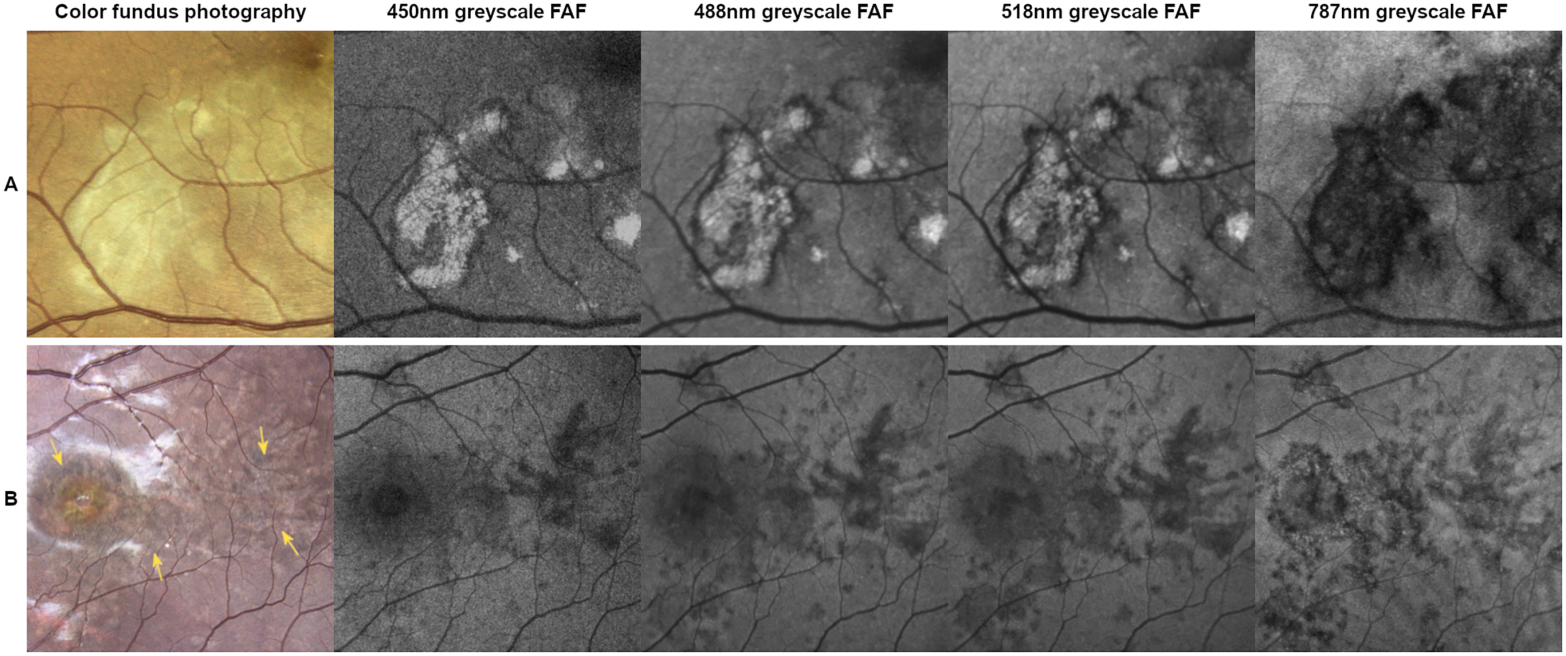
Comparison of inflammatory active and inactive lesions on color fundus photography (CFP) and 450 nm, 488 nm, 518 nm, and 787 nm excitation wavelength fundus autofluorescence (FAF) imaging. *Row A* = a 57-year-old woman with 61 months of disease duration. The subacute lesions present as “fuzzy” with poorly defined borders on CFP. On shorter wavelength FAF, they present a complex pattern of hyper- and hypoautofluorescent areas. Hyperautofluorescence is steadily decreasing whereas hypoautofluorescence is steadily increasing on longer excitation FAF wavelengths. *Row B* = a 25-year-old woman with 68 months of disease duration. Atrophic, inactive lesions appear dark with some pigmented areas on CFP (indicated by *arrows*) and hypoautofluorescent on all FAF modalities.

Across all FAF modalities, hyper-AF lesions had a higher probability of inflammatory activity, whereas hypo-AF lesions were less likely active (Table 3, Supplementary Tables S4, S5; Figs. 1, 3). OCT imaging, particularly in acute active stages, showed pronounced hyper-reflectivity within the outer nuclear layer (ONL), with blurred delineation of the outer retinal layers as well as disorganization, hyper-reflectivity, and disruption of the external limiting membrane and ellipsoid zone. Further findings included a loss of structural integrity of the outer retinal layers, resulting in atrophy (ONL to RPE), RPE thinning, and increased hypertransmission into the choroid.

**Table 3.** Clinical Activity of Lesions in Relation to FAF Characteristics

	Predicted Probability for Activity [95% CI]			
FAF Category	450 nm FAF	488 nm FAF	518 nm FAF	787 nm FAF
Hypo-AF	0.56 [0.25, 0.82]	0.51 [0.25, 0.82]	0.54 [0.25, 0.80]	0.61 [0.33, 0.84]
Iso-AF	0.59 [0.30, 0.83]	0.62 [0.32, 0.85]	0.59 [0.29, 0.84]	0.86 [0.45, 0.98]
Hyper-AF	0.86 [0.65, 0.95]	0.86 [0.64, 0.95]	0.87 [0.65, 0.96]	0.85 [0.62, 0.95]

95% CI, 95% confidence interval.

## Discussion

Our study provides a comprehensive analysis of APMPPE lesions on FAF and CFP, and indicates that FAF can aid assessing inflammatory activity and that it can be useful for identification and monitoring of clinically not visible lesions. Furthermore, our study shows that multimodal FAF encompassing a range of different excitation wavelengths allows for a more detailed phenotypic classification of APMPPE lesions. This is the first study to show a statistically significant association of hyper-AF with inflammatory activity in APMPPE.

To the best of our knowledge, APMPPE has not been analyzed by multimodal FAF with different excitation wavelengths so far. Furthermore, a statistical analysis of lesion characteristics in APMPPE has not yet been conducted. In our analysis, we found complex differences in APMPPE lesion characteristics in dependence of FAF excitation wavelength. Studies have been conducted comparing 488 nm FAF to 787 nm FAF in other posterior uveitis entities: for example, Li et al. found 787 nm FAF to be superior to 488 nm FAF in detecting subclinical lesions in PIC,^32^ and Battaglia Parodi et al. found 787 nm FAF to be a feasible tool for visualization of inflammatory lesions in MEWDS.^33^ The added value of using 450 nm FAF with spectrally resolved emission detection in APMPPE was shown recently by Wintergerst et al.^35^ These findings are in line with the results of our study. The differences in lesion characteristics on multimodal FAF can likely be explained by excitation of different retinal and choroidal layers and cellular components by different wavelengths.^19^ Whereas short-wavelength FAF mainly leads to fluorescence of lipofuscin and melanolipofuscin in the RPE, 787 nm FAF is mainly generated by melanin in the RPE and deeper lying choroid.^20^^–^^24^ This may also explain why the largest differences were found when comparing shorter wavelengths, such as 488 nm FAF, to the longest wavelengths like 787 nm FAF. Although in direct comparison of 450 nm FAF and 488 nm FAF, there was a trend toward more hyper-AF on 450 nm FAF differences when comparing 488 nm to 450 nm or 518 nm FAF were less prominent compared to those when comparing them to 787 nm FAF. The detailed origin of alterations in 787 nm FAF in APMPPE and its pathophysiological significance, however, need to be evaluated in further research. Multimodal FAF allowed for a detailed phenotypic characterization of APMPPE lesions, which might allow for a more detailed classification of APMPPE lesions for future disease staging.

In the literature, inflammatory active lesions secondary to APMPPE on FAF are mostly described as hypo-AF with a surrounding hyper-AF border. In the further course of active disease, lesions often appear in a mixed pattern of hyper- and hypo-AF.^7^^,^^14^^,^^17^^,^^36^ Considering that inactive lesions are described as hypo-AF, the distinguishing feature between active and inactive lesions is the presence of hyper-AF areas. In addition, in our analysis, lesions exhibiting hyper-AF were more likely active compared with the ones solely appearing hypo-AF across all autofluorescence modalities, therefore our statistical analysis further supports the association of hyper-AF lesions with clinical activity. Our study also illustrates that not all hyper-AF lesions can be considered clearly inflammatory active.

In our analysis, iso-AF lesions became less and hypo-AF lesions more frequent with increasing wavelength. Hence more lesions were visible overall on 787 nm FAF compared to short-wavelength FAF like 450 nm or 488 nm FAF (see Fig. 2). Furthermore, the extent of the displayed lesion area was often larger on 787 nm FAF compared to the other FAF wavelengths (see Fig. 1). A possible explanation for these findings could lie in the suggested choroidal inflammation involved in the pathophysiology of APMPPE.^7^^,^^8^ As the autofluorescence generated in 787 nm FAF originates from melanin not only in the RPE, but also the choroid,^22^^–^^24^ it may portray a different component of the pathophysiologic process in APMPPE compared with the other wavelengths. These findings are in line with research in other retinal diseases, such as recessive Stargardt disease, where changes on 787 nm FAF precede those on 488 nm FAF, with fleck profiles being larger on 787 nm FAF.^48^ A recent study suggesting an association of increased 787 nm FAF signal with oxidative stress could further support these assumptions.^49^ Taking the named aspects into account, 787 nm FAF could be used supplementary to CFP in clinical practice to diagnose and monitor the extent of chorioretinal lesions not visible funduscopically in patients with APMPPE. To portray the exact course of disease and determine disease stages, further basic research and longitudinal studies comparing 787 nm FAF to other means of imaging are warranted.

The strengths of our study are the inclusion of a multitude of FAF modalities and the relatively large sample size compared with other studies on APMPPE. Furthermore, our standardized, systematic qualitative evaluation of lesion characteristics and activity enabled comprehensive statistical analyses. Limitations of our study include that we did not use a quantitative approach for evaluation of lesion characteristics. Although a quantitative approach for evaluation would be preferable, it is more difficult to realize, as quantitative autofluorescence is to date only available for 488 nm FAF. Further, the evaluation of inflammatory activity was limited by the subjectivity of the grading, and lack of comparison with other imaging modalities, such as OCT-angiography, FA, or ICGA, which is reflected in the lower kappa value in comparison to the analysis of lesion characteristics. Because of the cross-sectional nature of our study, no causative conclusions can be drawn and no association with future course of disease can be made.

In conclusion, this first study on the use of multimodal FAF in APMPPE provides a detailed phenotypic description of lesions and its association with characteristics on CFP which could aid in disease and stage classification. As 787 nm FAF portrayed nearly all clinically not visible APMPPE lesions and often a larger extent of lesion area compared with other excitation wavelengths, this modality might be especially useful for clinical monitoring. However, clinical significance of funduscopically not visible lesions remains to be determined. Further, we found that hyper-AF is associated with inflammatory activity. FAF in general, and especially 787 nm FAF should therefore be routinely included in clinical management and studies.

## Supplementary Material

Supplement 1
