# Supplementary material for: Multi-Wavelength Autofluorescence Characteristics and Association With Inflammation in Acute Posterior Multifocal Placoid Pigment Epitheliopathy
Source: Invest Ophthalmol Vis Sci. 2025 Aug 26;66(11):62. doi: 10.1167/iovs.66.11.62 (PMC12395843; doi:10.1167/iovs.66.11.62)
Supplement: Supplement 1 [file iovs-66-11-62_s001.pdf]

Multi-wavelength Autofluorescence Characteristics and Association with Inflammation in Acute Posterior Multifocal Placoid Pigment Epitheliopathy

Robert P. Finger, Lennart J. Overbeck, Moritz Berger, Marie D. Just, Jana K. Koch, Jan H. Terheyden, Selina Foti, Matthias Schmid, Frank G. Holz, Thomas Ach, Maximilian W. M. Wintergerst

Supplementary Material

Supplemental Table 1. Characteristics on FAF in relation to CFP characteristics

		Predicted probability [95%CI]			
**CFP Category**	**FAF Category**	**450nm-FAF**	**488nm-FAF**	**518nm-FAF**	**787nm-FAF**
**Not visible**	Hypo-AF	**0.58** [0.36, 0.80]	**0.75** [0.58, 0.92]	**0.84** [0.71, 0.96]	**0.95** [0.91, 1.00]
	Iso-AF	**0.28** [0.17, 0.39]	**0.18** [0.07, 0.29]	**0.12** [0.03, 0.21]	**0.04** [0.00, 0.07]
	Hyper-AF	**0.14** [0.03, 0.25]	**0.07** [0.01, 0.13]	**0.04** [0.00, 0.08]	**0.01** [0.00, 0.02]
**White atrophic**	Hypo-AF	**0.44** [0.19, 0.68]	**0.70** [0.49, 0.92]	**0.73** [0.52, 0.93]	**0.87** [0.74, 0.99]
	Iso-AF	**0.34** [0.26, 0.41]	**0.21** [0.08, 0.34]	**0.19** [0.06, 0.33]	**0.10** [0.01, 0.19]
	Hyper-AF	**0.23** [0.05, 0.40]	**0.09** [0.01, 0.17]	**0.08** [0.00, 0.15]	**0.03** [0.00, 0.07]
**Complex**	Hypo-AF	**0.46** [0.16, 0.75]	**0.57** [0.27, 0.87]	**0.49** [0.18, 0.79]	**0.49** [0.18, 0.80]
	Iso-AF	**0.33** [0.23, 0.43]	**0.28** [0.13, 0.43]	**0.32** [0.20, 0.44]	**0.32** [0.20, 0.44]
	Hyper-AF	**0.21** [0.01, 0.41]	**0.14** [-0.01, 0.29]	**0.19** [0.00, 0.38]	**0.19** [0.00, 0.38]
**Greyish/ brownish**	Hypo-AF	**0.37** [0.15, 0.58]	**0.40** [0.17, 0.62]	**0.43** [0.21, 0.66]	**0.69** [0.49, 0.89]
	Iso-AF	**0.35** [0.31, 0.39]	**0.35** [0.30, 0.40]	**0.34** [0.27, 0.41]	**0.22** [0.10, 0.34]
	Hyper-AF	**0.28** [0.10, 0.47]	**0.26** [0.08, 0.43]	**0.23** [0.06, 0.39]	**0.09** [0.01, 0.17]

FAF= Fundus autofluorescence; CFP = Color fundus photography; AF=autofluorescent; CI= Confidence interval.

Supplemental Table 2.
Pairwise comparison of autofluorescence probabilities between FAF wavelengths within CFP categories.

	Estimate [p-value]			
	**Not visible**	**White atrophic**	**Complex**	**Greyish / brownish**
**450nm-FAF – 488nm-FAF**	**+0.7799** [0.0003]	**+1.1124** [0.0354]	+0.4747 [1.0000]	+0.1243 [1.0000]
**450nm-FAF – 518nm-FAF**	**+1.3279** [<0.0001]	**+1.2436** [0.0154]	+0.1386 [1.0000]	+0.2865 [1.0000]
**450nm-FAF – 787nm-FAF**	**+2.6864** [<0.0001]	**+2.1478** [<0.0001]	+0.1380 [1.0000]	**+1.3281** [<0.0001]
**488nm-FAF - 518nm-FAF**	+0.5480 [0.0905]	+0.1312 [1.0000]	-0.3361 [1.0000]	+0.1622 [1.0000]
**488nm-FAF - 787nm-FAF**	**+1.9065** [<0.0001]	+1.0354 [0.2344]	-0.3367 [1.0000]	**+1.2038** [<0.0001]
**518nm-FAF - 787nm-FAF**	**+1.3585** [<0.0001]	+0.9042 [0.4986]	-0.0006 [1.0000]	**+1.0416** [<0.0001]

FAF= Fundus autofluorescence; CFP = Color fundus photography.

Reported values correspond to log odds ratios. Positive values indicate higher odds for a higher autofluorescence level (hypo-autofluorescent < iso-autofluorescent < hyper-autofluorescent) in the first category compared to the second. In the example of the comparison of 450nm-FAF and 787nm-FAF this analysis revealed statistically significant more hyper-autofluorescence (compared to hypo- and iso-autofluorescence) for all CFP categories except complex. Significant values are indicated by bold letters.

Further comparisons of autofluorescence probabilities between CFP categories

On 518nm-excitation fundus autofluorescence (518nm-FAF) and 787nm-excitation fundus autofluorescence (787nm-FAF), not visible lesions were more likely hypo-autofluorescent compared to complex lesions, while complex lesions were more likely hyper- and iso-autofluorescent (Suppl. Tbl. 3). Comparison of white atrophic and complex lesions on 787nm-FAF showed a higher hypo-autofluorescence probability in white atrophic lesions and a higher iso- and hyper-autofluorescence probability in complex lesions. On 488nm-excitation fundus autofluorescence (488nm-FAF) and 518nm-FAF, white atrophic lesions were more likely to appear hypo-autofluorescent compared to greyish/brownish lesions, while greyish brownish lesions had a higher probability of being hyper- and iso-autofluorescent. Comparison of not visible to white atrophic, and complex to greyish/brownish lesions showed no significant differences.

Supplemental Table 3.
Pairwise comparison of autofluorescence probabilities between CFP categories within FAF wavelengths

	Estimate [p-value]			
	**450nm-FAF**	**488nm-FAF**	**518nm-FAF**	**787nm-FAF**
**Not visible - white atrophic**	-0.5766 [0.4847]	-0.2441 [1.0000]	-0.6609 [0.5801]	-1.1152 [0.1073]
**Not visible - complex**	-0.5016 [1.0000]	-0.8068 [0.8827]	**-1.6910** [0.0076]	**-3.0501** [<0.0001]
**Not visible - greyish/brownish**	**-0.8720** [0.0003]	-1.5277 [<0.0001]	**-1.9134** [<0.0001]	**-2.2303** [<0.0001]
**White atrophic - complex**	+0.0749 [1.0000]	-0.5628 [1.0000]	-1.0301 [0.6141]	**-1.9349** [0.0131]
**White atrophic - greyish/brownish**	-0.2955 [1.0000]	**-1.2836** [0.0012]	**-1.2525** [0.0029]	-1.1151 [0.0543]
**Complex - greyish/brownish**	-0.3704 [1.0000]	-0.7208 [1.0000]	-0.2225 [1.0000]	+0.8197 [0.8827]

FAF= Fundus autofluorescence; CFP = Color fundus photography.

Reported values correspond to log odds ratios. Positive values indicate higher odds for a higher autofluorescence level (hypo-autofluorescent < iso-autofluorescent < hyper-autofluorescent) in the first category compared to the second, and vice versa. Significant values are indicated by bold letters.

Supplemental Table 4.
Clinical activity of lesions in relation to FAF appearance (absolute values)

CFP Category	FAF Category	450nm-FAF	488nm-FAF	518nm-FAF	787nm-FAF
**Inactive**	Hypo-AF	27	46	54	91
	Iso-AF	65	45	39	2
	Hyper-AF	38	39	37	37
**Active**	Hypo-AF	31	38	44	99
	Iso-AF	61	55	43	15
	Hyper-AF	95	94	100	73

FAF= Fundus autofluorescence; CFP = Color fundus photography; AF=autofluorescent.

**Supplemental Table 5.**<br>Pairwise comparison of clinical activity between different FAF appearances				
	**Estimate [p-value]**			
	**450nm-FAF**	**488nm-FAF**	**518nm-FAF**	**787nm-FAF**
**Hypo-AF - Iso-AF**	-0.1381 [1.0000]	-0.4751 [1.0000]	-0.2215 [1.0000]	-1.3474 [0.5832]
**Hyper-AF - hypo-AF**	**+1.5925** [0.0013]	**+1.7670** [0.0001]	**+1.7020**[<0.0001]	**+1.2608** [0.0012]
**Hyper-AF - Iso-AF**	**+1.4544** [0.0006]	**+1.2919** [0.0046]	**+1.4805** [0.0019]	+0.0866 [1.0000]

FAF= Fundus autofluorescence; AF=autofluorescent.

Reported values correspond to log odds ratios. Positive values indicate higher odds for activity vs. inactivity in the first category compared to the second, and vice versa. Significant values are indicated by bold letters.

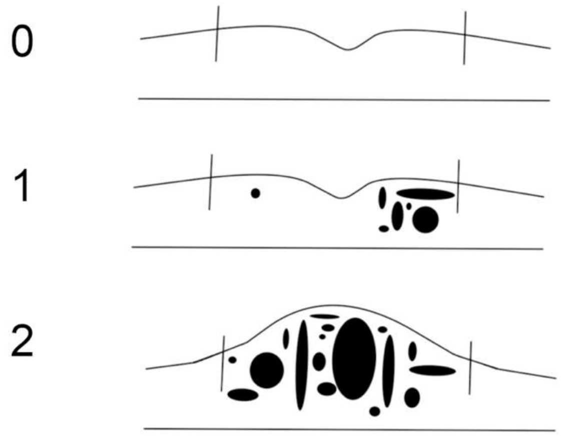

Supplemental figure 1. Classification of macular edema (ME) in optical coherence tomography images utilized in the analysis. (0) indicates absence of ME, (1) a presence of intra- or subretinal fluid without change of macular contour. ME of grade (2) is characterized by a presence of intra- or subretinal fluid with change of macular contour.

518nm greyscale FAF | **Color fundus photography**

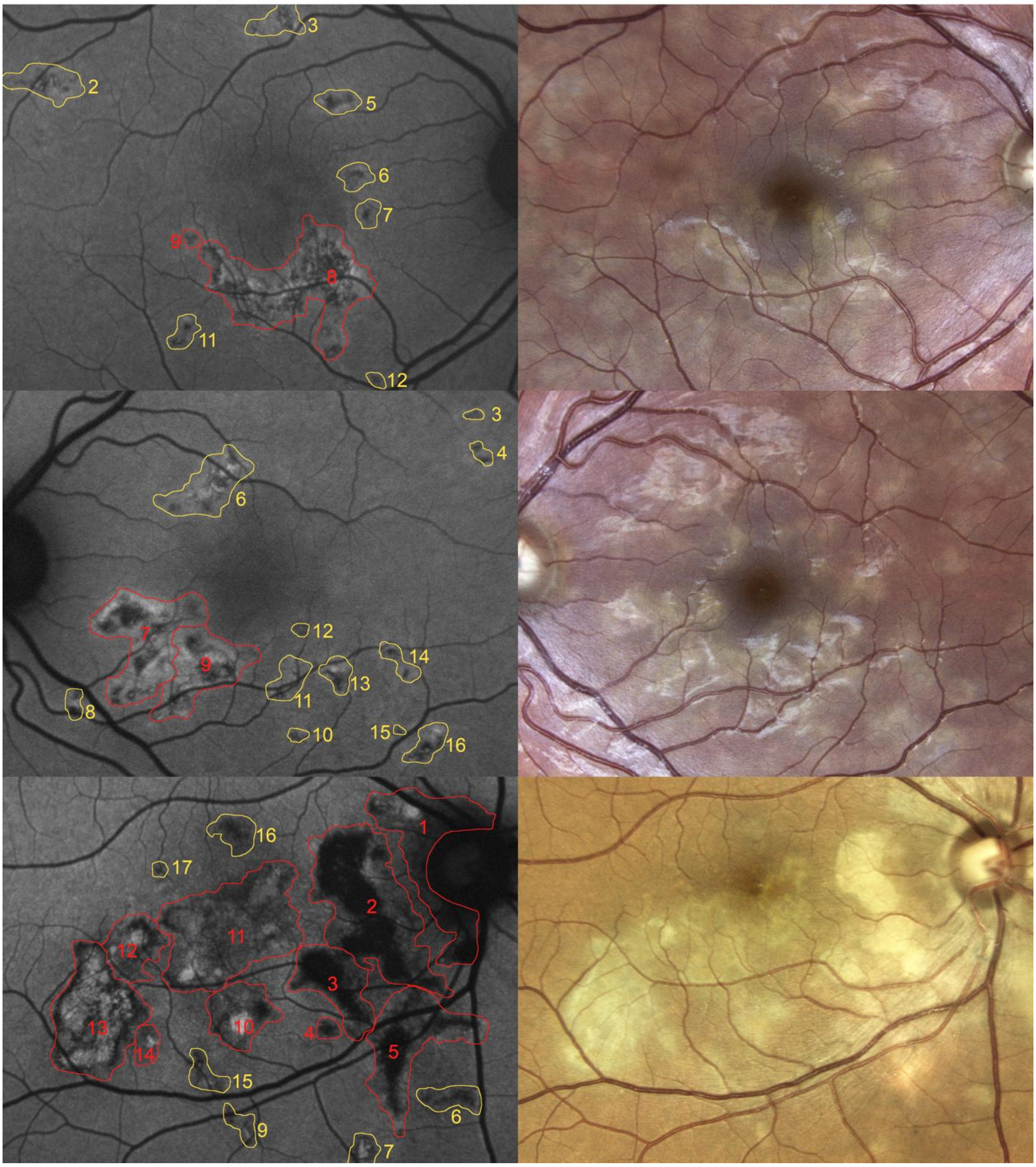

Supplemental figure 2. Exemplary illustration of assignment of lesion IDs on 518nm-excitation Fundus Autofluorescence (FAF) in Gnu Image Manipulation Program (left column). Color fundus photography images are shown in the right column for comparison. Brightness levels of FAF images were adjusted for better comparison. Lesion borders were demarcated on FAF in these examples for better visualization, missing lesion IDs are located outside of image borders. As lesions secondary to APMPPE are often coalescent and irregular, inter-rater agreement on assignment of lesion IDs was qualitatively assessed and a senior ophthalmologist was consulted in difficult cases (lesions marked in red), in order to guarantee comparability of the results for further analysis.
